# Host–pathogen interactions and subversion of autophagy

**DOI:** 10.1042/EBC20170058

**Published:** 2017-12-12

**Authors:** David G. McEwan

**Affiliations:** Division of Cell Signalling and Immunology, School of Life Sciences, University of Dundee, Dundee, U.K.

**Keywords:** autophagy, bacteria, host-pathogen interactions, infection, LC3, virus

## Abstract

Macroautophagy (‘autophagy’), is the process by which cells can form a double-membraned vesicle that encapsulates material to be degraded by the lysosome. This can include complex structures such as damaged mitochondria, peroxisomes, protein aggregates and large swathes of cytoplasm that can not be processed efficiently by other means of degradation. Recycling of amino acids and lipids through autophagy allows the cell to form intracellular pools that aid survival during periods of stress, including growth factor deprivation, amino acid starvation or a depleted oxygen supply. One of the major functions of autophagy that has emerged over the last decade is its importance as a safeguard against infection. The ability of autophagy to selectively target intracellular pathogens for destruction is now regarded as a key aspect of the innate immune response. However, pathogens have evolved mechanisms to either evade or reconfigure the autophagy pathway for their own survival. Understanding how pathogens interact with and manipulate the host autophagy pathway will hopefully provide a basis for combating infection and increase our understanding of the role and regulation of autophagy. Herein, we will discuss how the host cell can identify and target invading pathogens and how pathogens have adapted in order to evade destruction by the host cell. In particular, we will focus on interactions between the mammalian autophagy gene 8 (ATG8) proteins and the host and pathogen effector proteins.

## Basic mechanisms of autophagy

**Figure 1:**
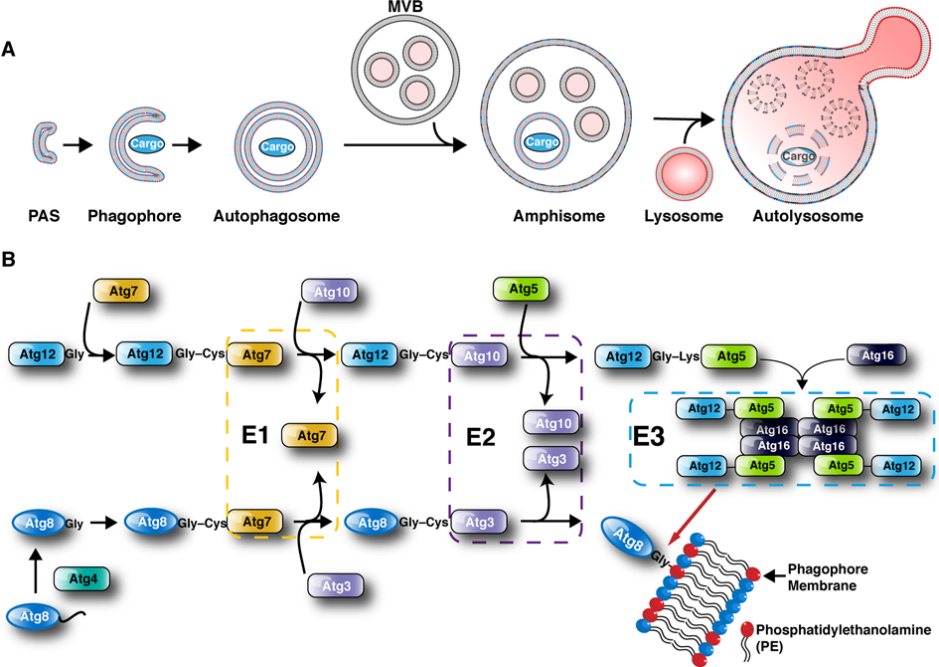
Autophagosome formation and Ubiquitin-like conjugation pathway (**A**) Autophagosome maturation occurs through several stages: initiation, elongation and termination. Autophagosomes form from a preautophagosomal structure (PAS), then mature to a phagophore that entraps cargo to a fully formed and sealed autophagosome prior to fusion with the endocytic compartment and termination at the lysosome (autolysosome). (**B**) Ubiquitin-like (UBL) conjugation machinery drive the formation of autophagosomes through the action of E1-like (autophagy gene 7 (ATG7)), E2-like (ATG3 and ATG10) and finally E3-like steps (ATG5–ATG12–ATG16 complex). This allows the conjugation of ATG8 (LC3 and γ-aminobutyric acid receptor associated proteins (GABARAP) in higher eukaryotes) to phosphatidylethanolamine (PE; red) that allows cells to target specific structures for degradation by selective autophagy.

Autophagy is the process by which cells can degrade intracellular content in the lysosome and recycle the basic constituents. This provides an intracellular pool of amino acids, lipids and basic building blocks that allow the cell to endure periods of stress, such as depletion of nutrients, oxidative stress or infection. The process of autophagy can be further subdivided into macroautophagy (the formation of a double-membraned vesicle, Figure 1A), chaperone-mediated autophagy (CMA) and microautophagy (direct substrate engulfment by the lysosome). Currently, there is little evidence for the role of microautophagy or CMA in tackling invading pathogens, so henceforth we will focus on macroautophagy (‘autophagy’).

## Initiation of autophagosome formation

In higher eukaryotes, autophagosomes form from phagophores (isolation membranes) that initiate at specific sites on the endoplasmic reticulum (ER) termed as omegasomes [1,2] but can also originate from the Golgi [3], mitochondrial outer membrane [4] and plasma membrane [5]. The formation of the isolation membrane at these sites is initiated under a variety of conditions, most notably through the inhibition of the master regulator mechanistic target of rapamycin (mTOR). Upon mTOR inhibition, the unc-51-like kinase 1/2 (ULK1/2) initiation complex, comprising ULK1/2, autophagy gene 13 (ATG13), FAK family kinase interacting protein of 200 kDa (FIP200) and ATG101 [6] is activated. This serves to recruit the autophagy class III phosphatidylinositol-3-kinase (PI3K) complex vacuolar protein sorting 34 (VPS34; PIK3C3), BECN1, PI3KR4, ATG14L and nuclear receptor binding factor (NRBF2), leading to phagophore expansion [7–11]. The local increase in phosphatidylinositol-3-phosphate (PI3P) allows the recruitment of PI3P-binding autophagy effector proteins including ZFYVE1/DFCP1, MTMR3 and WIPI2 [1,12–15]. WIPI2, a critical PI3P effector, serves as a platform for the recruitment of the ATG5–12–16 complex and is essential for the formation and expansion of the phagophore [14]. It is at this point the only known transmembrane ATG protein, ATG9, is recruited and acts as a potential membrane shuttling source for the growing isolation membrane. More recently, it was shown that both SRC tyrosine kinase and ULK1 can phosphorylate ATG9 at Tyr^8^ and Ser^14^ respectively, to regulate membrane delivery to the autophagosome [16].

## Ubiquitin-like conjugation machinery

The expansion of the phagophore to the fully formed and sealed autophagosome is driven by two ubiquitin-like (UBL) conjugation systems. First, ATG7 acting as an E1-like activating enzyme captures the UBL protein ATG12 and transfers it to ATG10, the E2-like enzyme. ATG12 is then attached, via an isopeptide bond, to the amino group of a lysine in ATG5. Second, the UBL ATG8 families comprising microtubule-associated protein light chain 3 (MAP1LC3A, MAP1LC3B and MAP1LC3C) and γ-aminobutyric acid receptor associated proteins (GABARAP, GABARAP-L1, GABARAP-L2) subfamilies [17–19], are C-terminally cleaved by ATG4 cysteine proteases (ATG4A–D), exposing an active glycine residue. This priming allows ATG7 to capture ATG8s in an ATP-dependent manner and transfer ATG8 to ATG3 (E2-like enzyme). Unlike ubiquitin, ATG8 proteins are not conjugated to lysine residues of target proteins but to phosphatidylethanolamine (PE) localized on the phagophore and can be attached to either the inner or outer isolation membrane (Figure 1B). This is catalysed by the ATG12–ATG15 conjugate that is now in complex with ATG16L1 [20,21]. The ATG5–12–16 complex acts as an E3-like enzyme that drives ATG8–PE conjugation and facilitates phagophore expansion [22] (Figure 1B).

## Selective autophagy

One advantage that autophagy has over the proteasomal pathway is its ability to sequester large molecular complexes, such as damaged organelles or intracellular bacteria, and deliver them for destruction in the lysosome. The process was once described as non-selective, where large portions of cytosol were encapsulated during starvation and a ‘random’ assortment of structures were recycled. However, it is now clear that cells can selectively target and degrade specific cargo. This is achieved primarily through ATG8/LC3/GABARAP interactions with autophagy receptor proteins that, along with the cargo, are also degraded. Examples include p62/SQSTM1 [23], NDP52 [24], OPTN [25], TAX1BP1 [26,27], FUNDC1 [28], NIX/BNIP3L [29], FAM134B [30] NBR1 [31]. These are usually distinct from autophagy adaptor proteins that also interact with LC3s/GABARAPs. The function of the adaptors in this instance is to drive the formation, transport and fusion of the autophagosomes. Autophagy adaptors are generally not degraded by the autophagy pathway [32]. Examples of autophagy adaptors include ULK1/2 [33], FYCO1 [34], TBC1D5 [35], PLEKHM1 [36] and TIAM1 [37]. However, there are instances where autophagy receptors can act as adaptors to facilitate the maturation of the autophagosome. For example, NDP52, classically known as a receptor targeting intracellular bacteria, has been shown to acts as an adaptor to regulate autophagosome maturation during *Salmonella enterica typhimurium* (*S. typhimurium*) clearance [38]. In addition, during Measles virus (MeV) infection, TAX1BP1 was shown to regulate autophagosome maturation [39]. Therefore, the lines between adaptors and receptors can, at times, be blurred. This raises new questions as to the mechanisms controlling the switch between adaptor and receptor functions.

Notably, what both receptors and adaptors have in common is the presence of an LC3 Interaction Region (LIR; also known as LC3 Interaction Motif (LIM) or Atg8 Interaction Motif (AIM)). With some notable exceptions, ‘atypical LIRs/LIMs’ of NDP52 [24], TAX1BP1 [26] and the dual LIR/UFIM (UFM1-Interaction Motif) in UBA5 [40], the majority of LIRs contain a core W/F/Y-x_1_-x_2_-L/V/I motif, where the side-chains of the bulky aromatic residue (W/F/Y) are placed deep inside a hydrophobic pocket 1 (HP1) on the Atg8/LC3/GABARAP surface, and side-chains of the hydrophobic LIR residues (L/V/I) occupy a second HP2 (reviewed in [41–43]). Other features that help define and identify LIR sequences include the presence of acidic and/or phosphorylatable serine/threonine residues N-terminal, to the core LIR/AIM that can stabilize the LIR–mATG8 interactions. The majority of LIRs are also found in unstructured regions between domains [44–46]. Recently, the LIR has been further refined with the identification of features that promote preferential interaction with GABARAP family of proteins, namely a [W/F]-[V/I]-x_2_-V or GABARAP Interaction Motif (GIM) [47]. This has added to the growing evidence of LC3 and GABARAP family-specific functions that are closely linked to their interaction with specific autophagy adaptors and receptors [19,36,48].

## Autophagosome maturation

The final stages of an autophagosome’s life cycle, where it is fully formed and contains cargo for destruction, is the fusion with the lysosome. This step is regulated by a large number of proteins including RAB7A [49], PLEKHM1 [36], homotypic fusion and vacuole protein sorting complex (HOPS) [36,50], ATG14 [51] and SNAREs (VAMP7, VAMP8, VTI1B, SNAP29 and STX17) [52–54] all of which mediate autophagosome-lysosome fusion to permit degradation of the cargo and inner autophagosomal membrane [55]. Cellular building blocks, such as amino acids and lipids are then recycled [56] and lysosomes are reformed [57]. This then serves as a major source of intracellular amino acids that allows cells and tissues to survive under stress condition.

One of the major functions of autophagy that has emerged in the past decade is its ability to act as an innate immune defence mechanism that targets intracellular pathogens and viruses. However, pathogens have developed mechanisms to evade and manipulate the system to allow them to survive, proliferate, escape and infect neighbouring cells. This ‘arms race’ between host and pathogen is a fascinating subject and, as further details emerge, will provide valuable insights into the role and regulation of the autophagy pathway in the innate immune system. This essay will cover some of the many strategies that both host and pathogens use to gain the ascendancy.

## Host defence mechanisms to target invading pathogens

### Detection

**Figure 2:**
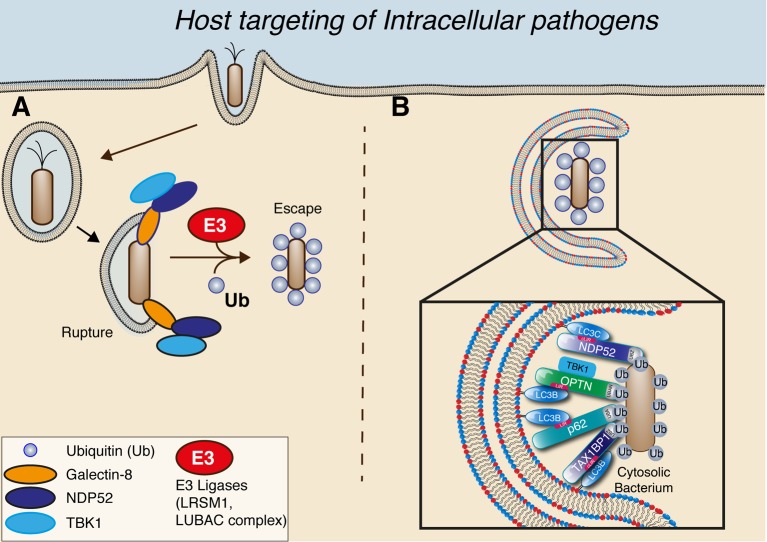
Methods for host targeting intracellular pathogens (**A**) Host cells sense damaged membranes through the action of galectin proteins, such as the case with galectin-8 in *Salmonella-*induced vesicle rupture (depicted). This brings the NDP52–TBK1 (TANK binding kinase 1) complex as first responders prior to the bacteria being coated in ubiquitin by E3-ligases (such as LRSM1 and LUBAC complex). (**B**) The ubiquitin coat acts as a ‘magnet’ to attract other autophagy receptor proteins such as OPTN, p62/SQSTM1 and TAX1BP1. The autophagy receptors interact with LC3 proteins through their LIR sequences and the ubiquitin coat via their respective ubiquitin binding domains (UBA, UBZ and UBAN). This allows the sequestration of cytosolic bacteria into autophagosomes and restriction of their proliferation.

Perhaps one of the most important questions for a host cell is how do they detect invading pathogens? What are the danger signs that trigger the innate immune response? Tellingly, one of the first ‘danger signs’ the cell recognizes is a damaged intracellular membrane. Notably, it was shown that the family of Galectins, which recognize glycans normally found on the luminal side of membrane-bound vesicles, are recruited to damaged membranes. Specifically, Galectins-3, -8 and -9, are recruited to sites of membrane rupture induced by *S. typhimurium* [58]. Galectin-3 is recruited to damaged lysosomes and acts as an ‘eat-me’ signal for lysophagy—the process of damaged lysosomes being degraded by the autophagy pathway [59]. Therefore, the Galectin proteins serve as a molecular surveillance mechanism to detect damaged or ruptured membranes that can be the first sign of infection. This is exemplified by Galectin-8 detection of ruptured pathogen vesicles. Infection of cells by the gram-negative bacterium, *S. typhimurium*, results in the bacterium residing within a *Salmonella*-containing vacuole (SCV) that matures similar to an endosome, progressively accumulating early endosome markers (such as EEA1 and Rab5) before maturing and obtaining Rab7 and LAMP1 late endocytic markers (reviewed in [60]). However, at the earlier time points during infection, the SCV can rupture through an ill-defined mechanism, thereby exposing the *S. typhimurium* and ruptured membrane vesicle to the host cytosol (Figure 2). This results in sensing the damaged membrane by Galectin-8 and recruitment of the autophagy-receptor protein NDP52, through a direct interaction between the carbohydrate recognition domain (CRD) of Galectin-8 and the C-terminal region of NDP52 [61,62]. NDP52 and Galectin-8 are the first responders to the damaged vacuoles [58] (Figure 2).

The recruitment of NDP52 is important to bring TANK binding kinase 1 (TBK1), which enhances the recruitment of WIPI2, itself essential for antibacterial autophagy [63]. Targeting of the cytosol-exposed bacteria by autophagy is enhanced by the presence of a dense ubiquitin coat that is mediated by several ubiquitin E3-ligases (Figure 2). Ubiquitin chains can be generated from any of the seven lysine residues present on ubiquitin as well as in a linear or ‘head-to-tail’ fashion via the N-terminal methionine [64–66]. The final conjugation step requires a specific E3-ligases that determines the chain type and target protein, leading to a variety of biological outcomes from regulation of signalling (K-63 linked) to degradation via the proteasome (K-48 linked).

The E3-ligase leucine-rich repeat and sterile α motif containing protein 1 (LRSAM1) was identified as an important ligase for the clearance of multiple intracellular bacterial pathogens such as *S. typhimurium, Listeria monocytogenes*, an internalized adherent invasive *Escherichia coli* and a *Shigella flexneri* strain *(ΔIcsB)* that can be targeted by autophagy [67]. The E3-ligase Parkin was shown to be required for the ubiquitin coat and autophagy-mediated clearance of *Mycobacterium tuberculosis* and *Salmonella typhi* [68–70], with mutations in *PARK2* that are associated with familial Parkinson’s disease giving rise to increased susceptibility of infection, typhoid fever and leprosy [68,70]. More recently, the linear ubiquitinase complex LUBAC has been shown to restrict cytosolic *S. typhimurium* proliferation by inducing xenophagy (selective removal of pathogens by autophagy) and local NF-κB signalling [71,72].

The addition of an ubiquitin coat can form distinct patches on the surface of the pathogen [72] that may aid the recruitment of host signalling molecules to specific regions of the invading pathogen. The action of the E3-ligases are, therefore, to generate a ubiquitin coat, which can be formed by several different linkages such as linear, K-63 and K-48 [73], all of which serve as ‘eat-me’ signals to recruit the host autophagy machinery. Interestingly, the predominantly cytosol-dwelling *S. flexneri* is able to escape this defensive mechanism through the action of a type III secretion system effector protein, IpaH1.4. IpaH1.4 is a secreted bacterial E3-ligase that ubiquitinates and degrades HOIP (E3 component of LUBAC) and suppresses NF-κB signalling [71,74], thereby promoting bacterial growth and escape.

## Delivery and destruction

After the cell has signalled danger (Galectins) and activated the ‘eat-me’ signal (ubiquitin coat), these serve to recruit the autophagy machinery and associated signalling molecules. This is primarily through the ubiquitin-binding regions and help link the ubiquitinated cargo (pathogen, damaged membrane) to the autophagosome that will ultimately surround, isolate and deliver the cargo for destruction. How the autophagosome forms around the pathogen is still unclear from a mechanistic standpoint, however, the receptor proteins themselves are able to interact with ATG8 proteins through the presence of a LIR/AIM/GIM. These receptors include p62/SQSTM1, OPTN, NDP52 and TAX1BP1 (Figure 2) and have been implicated in the growth restriction of intracellular *S. typhimurium* [25,26,63,75,76], *M. tuberculosis* [77], restriction of mutant *S. flexneri* (ΔIcsB) [24,78] and mutant *L. monocytogenes* [58,78,79]. Moreover, an intact autophagy pathway is required to restrict Group A *Streptococcus* (GAS) [80] and *Francisella tularensis* [81]. However, it is notable that most of these intracellular pathogens have evolved mechanisms for evading or manipulating this destructive pathway for their own benefit.

## Pathogen autophagy avoidance or subversion mechanisms

Bacterial pathogens are expert manipulators of intracellular trafficking pathways. Therefore, it comes as no surprise that they have adapted to the host innate immune defence mechanisms for dealing with infection. This can happen in multiple ways but with respect to the autophagy pathway, they can inhibit or block autophagy, or subvert the machinery to provide a stable pathogen-inhabited compartment to proliferate and eventually disseminate from.

**Figure 3:**
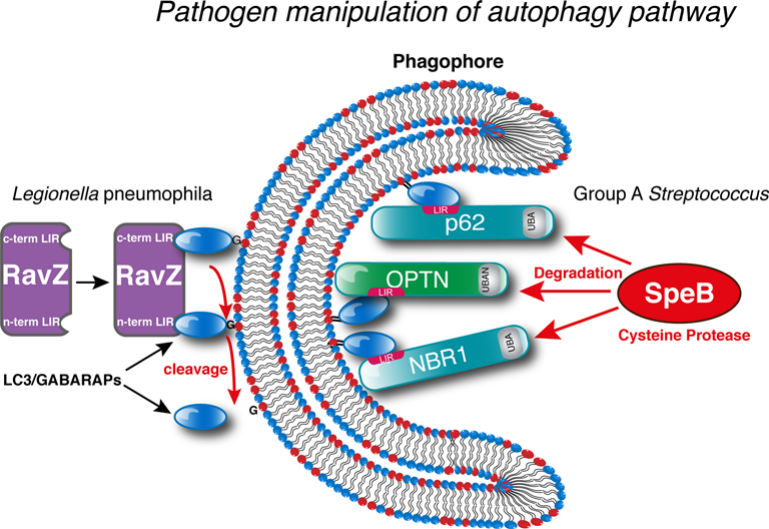
Methods for pathogen manipulation of host autophagy machinery Pathogens such as *L. monocytogenes* secrete effector proteins that irreversibly cleave ATG8 proteins preventing their reconjugation to the autophagosome and inhibiting their autophagy-mediated clearance. Other strategies include the cysteine protease SpeB secreted by GAS which efficiently degrades the autophagy receptor proteins p62/SQSTM1, NBR1 and NDP52 to prevent targeting by selective autophagy.

For example, wild-type *S. flexneri*, after invasion of the host cell, rapidly breaks free of its vacuole to gain access to the cytosol. Once in the cytosol, this activates the Gal8-NDP52-TBK1 pathway and the host ubiquitin conjugation machinery [58,71,78,82]. However, *Shigella* is able to shield itself from detection and removal by autophagy through the actions of a secreted effector protein, IcsB, which surrounds the bacteria and prevents recruitment of ATG5 and the NDP52-LC3 machinery [82,83]. Another pathogen that directly targets the host autophagy machinery is the M1T1 clone GAS serotype. M1T1 GAS secretes the cysteine protease SpeB that targets host autophagy receptor proteins NDP62, p62 and NBR1 for degradation and can actively replicate in the host cytosol (Figure 3), unlike other serotypes such as the M6 clone [84].

The Gram-positive bacterial pathogen, *L. monocytogenes*, is a professional cytosol-dwelling bacterium that has evolved multiple ways of avoiding or manipulating the autophagy pathway in order to survive. Ordinarily, *L. monocytogenes* utilizes a cholesterol-dependent pore-forming toxin, listeriolysin O (LLO), to mediate escape from an intracellular phagosome after uptake by the cell [85,86]. However, under conditions of inefficient LLO expression or activity, *L. monocytogenes* reside within spacious *Listeria*-containing phagosomes (SLAPs) [87]. These compartments resemble autophagosomes (LC3 and LAMP1 positive) and *L. monocytogenes* are able to proliferate, albeit more slowly, compared with the cytosolic bacteria [87]. The listerial virulence factor ActA, which is essential for Arp2/3 complex, Ena/VASP recruitment, subsequent actin-mediated intracellular motility and cell-to-cell dissemination and helps disguise *L. monocytogenes* from host-mediated autophagic recognition and destruction [79]. In addition, *L. monocytogenes* can ‘cloak’ itself from detection by host autophagy machinery by utilizing the effector protein InlK to recruit host major vault protein (MVP), thereby increasing bacterial survival in infected cells [88].

Other intracellular pathogens can manipulate the host autophagy machinery to help the supply of nutrients and membranes. For example, wild-type *S. typhimurium* that resides in the vacuole can utilize the autophagy machinery to repair the damaged SCV [89].

*Staphylococcus aureus* can exploit the autophagy machinery to form its replicative niche, which resembles an autophagosome, i.e. they are double membraned and stain positive for LC3 but do not mature to LAMP-positive autolysosomes [90]. *S. aureus* can also decrease autophagic flux of cells through expression of IsaB effector proteins through an, as yet, undefined mechanism [91].

Infection of host cell by MeV induces autophagic flux that is essential for viral replication within the cell [92]. Interestingly, MeV utilizes autophagy receptor proteins NDP52 and TAX1BP1, but not OPTN or p62/SQSTM1, possibly through a direct interaction with viral effector proteins, for the maturation of a subset of MeV-containing autophagosomes required for optimal MeV replication [39].

## Mimicking the LIR to subvert host machinery

Perhaps one of the more interesting themes to emerge from the host–pathogen interactions is the emerging evidence that certain pathogen effector proteins contain LIRs to aid the subversion of the host autophagy machinery. This has been demonstrated now for both viruses and bacterial effectors. For example, the influenza A virus (IAV) Matrix 2 (M2) ion channel protein blocks autophagosome-lysosome fusion [93] and recruits LC3 to the plasma membrane through an LIR motif present on the cytoplasmic tail of the M2 protein [94]. The recruitment of LC3 in this manner is essential for viral budding and transmission [94].

One of the best examples of bacterial effectors manipulating autophagy through LIR-mediated interactions is the *Legionella pneumophila* effector protein RavZ. RavZ acts to selectively and irreversibly deconjugate LC3/GABARAPs from PE on the autophagosomal membrane through cleavage of the amide bond between the C-terminal glycine of LC3 and the preceding aromatic residue (Figure 3). This results in an irreversibly cleaved LC3/GABARAP protein that cannot be reconjugated to the autophagosomal membrane [95]. RavZ is directed towards the autophagosomal membrane through PI3P-dependent interaction and curvature-sensing motifs [96] where it can subsequently interact with LC3 through two LIR motifs located at the N- and C-termini [97,98] (Figure 3). Interestingly, it seems that only the N-terminal LIR is required for the proteolytic cleavage of LC3 from the membrane [98] but the presence of the second, C-terminal LIR may serve to increase the binding affinity for membrane-bound LC3 and facilitate the correct orientation for LC3–PE cleavage [97]. This raises an intriguing question as to the function of removing LC3/GABARAPs from the surface of autophagosomes. Removal of LC3/GABARAPs from the autophagosomal membrane by RavZ results in the inhibition of autophagy [95]. However, as *L. pneumophila* replicates in a ‘*Legionella*-containing vacuole’ (LCV) within macrophages, could the action of RavZ be a defence mechanism to prevent autophagosome fusion with the LCV, or perhaps a mechanism to ensure a plentiful supply of intracellular membrane for the proliferation and eventual dissemination of as *L. pneumophila*?

Notably, a database of viral proteins from over 16000 viral sequences and 2500 viral species, revealed a large number of potential LIR sequences contained within the viral proteins [99]. For example, a potential LIR was identified in the HIV-1 protein Nef which has previously been shown to inhibit autophagosome maturation and to colocalize with LC3 and BECN1 to protect HIV-1 from autophagy-mediated clearance [100,101]. Obviously, not all 15000 identified potential LIRs will be *bona fide* and functional sequences that are important for the viral life cycle. However, this does raise an important question as to whether the manipulation of host autophagy machinery can be exploited in a more general way by viral proteins through direct interaction with LC3 and GABARAP proteins.

## Concluding remarks

In the constant ‘arms race’ between the ever evolving host–pathogen interactions, it is becoming increasingly clear that targeting the host autophagy machinery is high on the agenda for pathogens. This manipulation allows them to avoid destruction, to form a pathogen inhabited compartment, repair their replicative niche or just as a delivery mechanism to ‘order in’ nutrients that they require to survive, proliferate, escape and re-infect. As more details emerge of how pathogens achieve this, we will undoubtedly gain new insights into the regulation of autophagy that may open new avenues for therapeutic intervention, not only for the treatment of infectious diseases but also in the fight against cancer, neurodegenerative and metabolic diseases.

## Summary

- Macroautophagy is a degradative pathway for the delivery of a range of substrates inside a double-membraned vesicle (autophagosome) for destruction in the lysosome.
- Autophagy is an essential component of the innate immune system’s defence against invading pathogens.
- Cells are alerted to intracellular pathogens by the presence of damaged membranes. This helps to recruit E3-ligases to generate the ‘eat-me’ signal.
- Autophagy receptor proteins such as p62/SQSTM1, NDP52, TAX1BP1 and OPTN can then target ubiquitinated intracellular pathogens for destruction by autophagy.
- Bacteria and viruses have evolved novel methods to combat the autophagy-based defence mechanisms, such as inhibition of autophagosome formation, degrading autophagy receptors or removing LC3/GABARAPs from the autophagosomal membrane.
- Understanding how pathogens evade or manipulate autophagy will shed light on potential opportunities for therapeutic intervention to combat infection.

## Abbreviations

AIM: Atg8 interaction motif
ATG: autophagy gene
BCL-2: B-cell lymphoma 2
CMA: chaperone-mediated autophagy
EEA1: Early endosome antigen 1
FAK: Focal Adhesion Kinase
GABARAP: γ-aminobutyric acid receptor associated protein
GAS: Group A *Streptococcus*
GIM: GABARAP interaction motif
HOIL-1: Heme-oxidized IRP2 ubiquitin ligase 1
HOIP: HOIL-1-interacting protein
HP: hydrophobic pocket
LCV: *Legionella*-containing vacuole
LIM: LC3 interaction motif
LIR: LC3-interaction region
LLO: listeriolysin O
LUBAC: linear ubiquitin chain assembly complex
MAP1LC3 (LC3): Microtubule-associated proteins 1A/1B light chain 3B
MeV: Measles virus
mTOR: mechanistic target of rapamycin
M2: matrix 2
NBR1: next to *BRCA1* gene 1
NDP52: nuclear dot protein 5
NF-κB: Nuclear factor -kappa-B
NRFB2: nuclear receptor binding factor
OPTN: optineurin
PAS: preautophagosomal structure
PE: phosphatidylethanolamine
PI3P: phosphatidylinositol-3-phosphate
PLEKHM1: pleckstrin homology domain containing family M member 1
SCV: *Salmonella*-containing vacuole
SNARE: Soluble NSF Attachment Protein Receptor
SQSTM1: sequestosome-1
STX17: Syntaxin17
TAX1BP1: Tax1-binding protein 1
TIAM1: T-cell lymphoma invasion and metastasis 1
TBC1D5: Tre-2/Bub2/Cdc16 1 domain family member 5
TBK1: TANK binding kinase 1
UBL: ubiquitin-like
UFIM: UFM1-interaction motif
UFM1: Ubiquitin-fold modifier 1
ULK1/2: unc-51-like kinase 1/2
WIPI: WD repeat domain phosphoinositide-interacting protein
